# A Study of Awareness about HIV/AIDS Among Senior Secondary School Children of Delhi

**DOI:** 10.4103/0970-0218.42063

**Published:** 2008-07

**Authors:** P Lal, Anitha Nath, S Badhan, Gopal K Ingle

**Affiliations:** Department of Community Medicine, Maulana Azad Medical College, New Delhi, India

## Introduction

School children of today are exposed to the risk of being victims of HIV/AIDS - which was quite unknown to their predecessors a few decades ago. The epidemic of HIV/AIDS is now progressing at a rapid pace among young people. Studies have reported that young people form a significant segment of those attending sexually transmitted infection (STI) clinics and those infected by HIV.(1) Programme managers and policy makers have often recommended that schools can act at the center point for disseminating information and education on HIV/AIDS. Hence school education has been described as a ‘social vaccine’, and it can serve as a powerful preventive tool. In India, there is a wide gap between the inputs in the HIV/AIDS curriculum for schools and the actual education that is imparted.(2) As children are a valuable resource for the future of a country, it is imperative that they be equipped with ample amount of information so as to protect themselves and their counterparts from falling a prey this still-an-incurable killer disease. With this background, the present study was conducted with the following objectives: (i) To assess the awareness of school children regarding HIV/AIDS; (ii) to provide suggestions for school AIDS education.

## Materials and Methods

The present study was undertaken by the Department of Community Medicine, Maulana Azad Medical College and Harbans Kaur Memorial Trust (HKMC), a non-governmental organization (NGO) and a partner of programme implementation by Delhi State AIDS Control Society (DSACS). Out of 1689 senior secondary schools in South Delhi area, 60 schools (3.5%) had been allotted to HKMC Trust by DSACS for carrying out school AIDS education programmes, in which there were 48 government schools (23 boys school, 23 girls and 2 co-educational) and 12 private co-educational schools. A total of 2592 students belonging to Classes IX to XI in these schools participated in the study. The response rate of students was 100%. The study was conducted over a period of 3 months from 1^st^ August 2005 to 31^st^ October 2005. The students were administered a pre-designed proforma, which included multiple choice questions. Written consent was obtained from the school principals after explaining the purpose of the study to them. Data were entered and analyzed using SPSS version 13.0 by means of simple comparison of proportions.

## Results and Discussion

In the present study, majority of the students (74.9%) belonged to the age group of 15-17 years. The mean age was 15.8 ± 0.8 years (see Table 1). Most of them (60%) were females. All the students had heard of HIV/AIDS although only 51.4% were able to write the full form of AIDS and only 19.9% were able to write the full form of HIV, as shown in given Table 1. This is comparable to the observations of a study carried out amongst the secondary school students in Haryana and Jamnagar.(3,4) However, a baseline assessment on HIVAIDS awareness amongst 250 Nigerian school students revealed that only 5% were able to expand HIV and AIDS.(5)

**Table 1:** Age and sex wise distribution of study subjects and awareness regarding HIV/AIDS

Variable	Male(%)	Female (%)	Total (%)
Age (years)			
<14	172 (16.6)	262 (16.9)	434 (16.7)
15-17	727 (70.1)	1216 (78.2)	1934 (74.9)
>18	138 (40.1)	77 (4.9)	215 (8.4)
Total	1037 (40.1)	1555 (59.9)	2592 (100)
Awareness regarding HIV/AIDS*			
Heard of HIV/AIDS (yes)	1037 (100)	1555 (100)	2592 (100)
Wrote full form of AIDS†	404 (39.5)	920 (59.3)	1324 (51.4)
Wrote full form of HIV	114 (11.2)	400 (25.8)	514 (19.9)
Mode of transmission*			
Sexual intercourse	489 (47.8)	751 (48.4)	1240 (48.2)
Blood transfusion	252 (24.6)	549 (35.4)	801 (31.1)
Sharing needles/syringes	397 (38.8)	747 (48.1)	1144 (44.4)
Mother to baby**	146 (14.3)	456 (29.3)	602 (23.4)
Knowledge about AIDS being preventable	732 (71.6)	1125 (72.4)	1857 (72.1)
Method of prevention*			
Condom**	155 (21.2)	122 (10.8)	277 (14.9)
Safe blood	6 (0.8)	18 (1.6)	24 (1.3)
Disposable syringes	20 (2.8)	38 (3.4)	58 (3.1)
Awareness about availability of treatment for HIV/AIDS	57 (7.8)	152 (13.5)	736 (28.6)

* Responses are not mutually exclusive

† Indicates significant difference, *P* < 0.05

Only 48.2% of the students could name sexual route while 44.4% named sharing of syringes and needles as a mode of transmission [Table 1]. Similar findings were observed in a study done amongst 2400 secondary school students from Mumbai, in which only 50% of students knew about the sexual route of transmission. In our study, gaps were seen in the awareness about other modes of transmission wherein only 31.1% and 23.4% cited blood transfusion and mother to baby transmission as routes of transmission, respectively. Low levels of knowledge about general aspects and transmission of HIV/AIDS have also been observed amongst secondary school students in Kolkata.(6) Studies conducted in other countries have reported higher levels of knowledge regarding transmission routes.(7,8) This difference in knowledge could be attributed to early appearance of disease in these countries.

In the present study, only 72% of students were aware about HIV/AIDS as being preventable. Moreover, awareness about the different methods of prevention was rather low. Only 14.9% had knowledge about condoms as a means of protection, which awareness was significantly higher amongst boys. Higher levels of awareness have been observed amongst school children of Haryana.(9) Studies conducted in other countries have also reported high awareness levels regarding condom for HIV/AIDS prevention.(10–12) Only 28.6% knew about the availability of drugs for HIV/AIDS. This was similar to the observation made amongst a group of secondary school students belonging to Udupi district in Karnataka, in that only 24.3% were aware about the existence of drugs while a slightly higher number of school students (34%) in Mumbai knew about the availability of antiretroviral drugs.(13,14)

With regard to the sources of information about HIV/AIDS, 79.6% of the students mentioned that television and radio were the main sources of information to them. Likewise, a majority (62.7%) of senior secondary students belonging to a government school in Chandigarh reported that they derived most of the information from TV and radio.(9) In our study, only 9.5% of children had heard about HIV/AIDS through their respective school programmes. This finding suggests that school AIDS education should be strengthened further in schools. As much as 8.6% had obtained information from print media, whereas for 2.3%, friends remained the source of information. These findings imply promoting television as a significant source of information. A greater involvement of print media can also be a cost-effective measure. Friends can also be made instrumental in spreading information through frequent motivation. Published literature indicates that peer education has a significant impact in reducing risk behavior.(15)

In our study, a majority (77.8%) of students had a favorable attitude towards People Living with HIV/AIDS (PLWHA), stating that such patients should be allowed to pursue/continue studies or allowed to work in common work places. However, this favorable attitude towards HIV positive patients was not observed among college students in Nashik.(16) About 51.6% of students in the present study felt that PLWHAs must be hospitalized while 33.3% were in favour of home care.

The findings in the present study reiterate the need for re-enforcing school AIDS education. Significant changes have been observed between pre-test and post-test knowledge and awareness levels through school HIV/AIDS education programmes in different regions.(17–19) While the teacher plays a pivotal role in imparting education, the use of multi-pronged methods such as films, group discussions, dramas, puppet shows and role-plays must be incorporated. There is a strong need that school education must directly address stigmatizing attitudes about HIV/AIDS, gaps in HIV/AIDS knowledge and awareness of HIV-related health resources.
